# Early versus delayed enteral nutrition for neonatal hypoxic-ischemic encephalopathy undergoing therapeutic hypothermia: a randomized controlled trial

**DOI:** 10.1186/s13052-022-01342-2

**Published:** 2022-08-15

**Authors:** Ya Hu, Feng Chen, Xinyu Xiang, Fang Wang, Ziyu Hua, Hong Wei

**Affiliations:** 1 Department of Neonatology, Children’s Hospital of Chongqing Medical University (The institution is also validated by Ringgold as ‘Chongqing Medical University Affiliated Children’s Hospital’), Chongqing, China; 2 Ministry of Education Key Laboratory of Child Development and Disorders, Chongqing, China; 3 National Clinical Research Center for Child Health and Disorders, Chongqing, China; 4 China International Science and Technology Cooperation base of Child Development and Critical Disorders, Chongqing Key Laboratory of Pediatrics, Chongqing, China

**Keywords:** Enteral nutrition, Hypoxic-ischemic encephalopathy, Therapeutic hypothermia, Neonate, Feeding intolerance

## Abstract

**Background:**

The practice of therapeutic hypothermia (TH) is widely used for neonatal hypoxic-ischemic encephalopathy (HIE) despite its corresponding feeding strategies are still controversial. This randomized controlled trial (RCT) demonstrated to evaluate the effect of early vs. delayed enteral nutrition on the incidence of feeding intolerance (FI) and other association during TH.

**Methods:**

This single center, parallel-group, and no-blinded RCT was processed in a level III, and academic neonatal intensive care unit. Infants who were diagnosed with HIE and undertaken TH from September 2020 to August 2021 were enrolled. Participants were randomized to receive enteral nutrition either during TH/rewarming (early enteral nutrition, EEN) or after TH (delayed enteral nutrition, DEN) according to a recommend enteral feeding protocol. All data were analyzed using SPSS 26.0 software with a *p*-value< 0.05 was considered statistically significant.

**Results:**

Ninety-two infants were enrolled after randomization, but 12 (13.04%) cases including 3 (3.26%) deaths were excluded from eventually analyzed, who did not initiate or discontinue the intervention. 80 cases (42 and 38 in the EEN and DEN group, respectively) who completed the interventions were eventually analyzed. Besides initial time of enteral feeds, two groups had processed the same feeding method. Total 23 (25.0%) cases developed FI, and no difference of morbidity was found between two groups (23.4% vs 26.7%, *p* = 0.595; Log Rank, *p* = 0.803). There was no case died or developed late-onset bloodstream and no difference of the incidence of hypoglycemia or weight gain was found (*p* > 0.05). The percentage of infants who had not reaching the goal of full enteral feeding volume between the two groups was similar (21.43% vs 23.68%, *p* = 0.809). The average time of parenteral nutrition, reaching full enteral feeds and hospital stay were shorter in the EEN group compared with the DEN group with significant differences (8.81 ± 1.67 vs 10.61 ± 2.06 days, *p* < 0.001; 9.91 ± 1.88 vs 12.24 ± 2.50 days, *p* < 0.001; 12.55 ± 4.57 vs 16.47 ± 5.27 days, *p* = 0.001 respectively).

**Conclusions:**

Compared with delayed enteral nutrition, introduction of early enteral nutrition according to a recommend feeding strategy for neonatal HIE undergoing TH may be feasible and safe.FI is frequent in this high-risk group of infants which should not be ignored during feeding process.

**Trial registration:**

The Chinese Clinical Trial Registry,ChiCTR2000038193, 2020-9-13, https://www.chictr.org.cn.

## What is already known on this topic?

- ► The practice of TH is widely used for neonatal HIE despite its corresponding feeding strategies are still controversial.
- ► There is few study suggests enteral feeds during TH is safe without significant increase in complications but lack of evidence from randomized controlled trial.
- ► Most of the discussions about the safety of enteral feeding during TH mainly focused on the incidence of NEC, while feeding intolerance (FI) was not mentioned in any study.

## What this study adds?

- ► Early enteral nutrition during TH is not increasing the complications compared with the delayed enteral nutrition after TH in neonatal HIE.
- ►According to a recommend enteral feeding protocol, early enteral nutrition can shorten the time of parenteral nutrition, reaching full enteral feeds and hospital stay.
- ► Introduction of early enteral nutrition carefully may be a better choice for neonatal HIE undergoing TH according to a recommend feeding method.
- ► Our study is the first RCT to evaluate the effect of early vs. delayed enteral nutrition (EEN vs. DEN) on the incidence of feeding intolerance.

## Introduction

With more application of Therapeutic hypothermia (TH) for moderate to severe hypoxic-ischemic encephalopathy (HIE) in neonatal period, some studies have focused on infants’ nutrition strategies during TH [1–7]. Among the United Kingdom of Great Britain and Northern Ireland (UK) neonatal intensive care units (NICUs) only 31% of the responding units had feeding guidelines in HIE and 37% of them are used to providing intravenous dextrose fluids without feeds [1]. In China, many NICUs common delay enteral nutrition due to the risk of developing necrotizing enterocolitis (NEC) [8–10]. In contrary, other NICUs often practice minimal enteral nutrition [2, 3, 6] during TH in addition to parenteral nutrition (PN) [1].

To our knowledge, these differences in practice are mainly due to concerns about the safety of early enteral nutrition during TH. The commencement and progress of enteral feeds during TH is still an unresolved topic. The initiation of enteral feeding is often delayed in newborns undergoing TH due to the concern that early enteral feeding may not be tolerated and may be implicated in the pathogenesis of necrotizing enterocolitis (NEC) [11]. However, the lack of enteral feeds may hinder the functional maturation of the gastrointestinal tract, and the prolonged use of parenteral nutrition is associated with an increased risk of systemic infection due to the impairment of immune cell functions [12, 13]. Although a newest large retrospective study of 6030 babies about nutritional management by Gale C et al. [13] has found enteral feeds during TH (instead of after) may decrease the risk of NEC, and support introduction of enteral feeding during TH, but still lack of high-quality studies on this topic. Another few studies have found that early enteral feeds can be tolerated by neonates with HIE receiving TH without a significant increase in feeding complications [1–5]. However, all studies on this topic still have showed several limitations such as the feeding protocols were not standardized and no one was a randomized controlled trial (RCT) [6].

On the other hand, most of the discussions about the safety of enteral feeding during TH mainly focused on the incidence of NEC, while feeding intolerance (FI) was not mentioned in any study [1–6]. Although the most serious feeding complication for enteral feeding during TH is NEC, but it has been proven that the incidence of NEC is very rare with a morbidity from 0 to 0.5% [3, 6, 13] in infants undergoing TH. For this reason, only chosen the incidence of NEC as a primary outcome in such studies to evaluate the safety of enteral nutrition during TH may be not suitable. In fact, FI occurs in about one-third of critically ill patients [14] and is a common problem in intensive care units worldwide [15, 16]. One of the risk factors for the development of FI is the malfunctioning enteric system and oxygenation [17], which is much common among neonatal HIE and we have observed that FI is very frequent in clinic practice. So FI should not be ignored as an important indicator to evaluate the safety of enteral nutrition during TH. Therefore, to ensure the appropriate feeding strategy is performed, it is necessary to further identify the effect of early vs. delayed enteral nutrition (EEN vs. DEN) with a RCT on the incidence of FI.

In this randomized controlled trial, we investigated all newborns with moderate to severe HIE who underwent TH at a pediatric tertiary hospital during a 1-year period. We try to evaluate the effect of early vs. delayed enteral nutrition (EEN vs. DEN) on the incidence of feeding intolerance and other association during therapeutic hypothermia between enteral feeding and outcomes such as late-onset bloodstream infection and hypoglycemia for patients with neonatal hypoxic-ischemic encephalopathy (HIE) undergoing TH.

## Materials and methods

### Study design and participants

This single center, non-blinded, parallel-group randomised controlled trial (RCT) study was conducted in the Department of Neonatology at Children’s Hospital of Chongqing Medical University, which has 300 beds and admits approximately 9000–10,000 newborns each year without a delivery room in Chongqing, China between September 2020 to August 2021.

In the present study, we enrolled neonates born at 35^0/7^ to 42^6/7^ weeks of gestation who required admission to the neonatal diagnosis center of Children’s Hospital. The RCT was performed to explore whether the incidence of FI during hospital stay differed between neonates with early enteral nutrition (EEN) [initiation of enteral feeds during therapeutic hypothermia/rewarming] and those with delayed enteral nutrition (DEN) [initiation of enteral feeds after therapeutic hypothermia/rewarming] according to a feeding guideline over 1 year. Follow-up was performed for some patients who were discharged early without clinicians` advice to achieve total enteral nutrition. Telephone follow-up was conducted 7 days after their discharging to understand the short-outcome.

### Inclusion and exclusion criteria

Upon admission to our NICUs and in the course of research, all neonates were screened for eligibility for inclusion or exclusion by one independent observer.

Inclusion criteria: (1) Meet the criteria for moderate-severe HIE diagnosis according to Levene’s modification of the Sarnat [18] and undergoing TH [19]; (2) Gestational age at birth ≥35 completed weeks, and birth weight ≥ 2.0 kg; (3) Admission age not exceeded 6 hours of birth.

Exclusion Criteria: (1) Enteral feeding was initiated prior to randomization; (2) Considered as congenital malformation or hereditary metabolic diseases which may affect enteral feeding such as Hirschsprung’s disease or Phenylketonuria prior to randomization; (3) Declined to participate the study for the parents` decisions.

### Data collection

We got reliable medical data from the medical data platform of Children’s Hospital of Chongqing Medical University. For each patient, the study data were recorded in an electronic case-report form. Collected data included demographic characteristics (including maternal risk factors and infantile factors), Apgar scores, complications, laboratory results (blood gas data, traditional markers of inflammation at admission), adjuvant therapy, feeding protocol and clinical outcomes. Data of follow-up only for infants who did not reach full enteral feeds included current feeding status and whether gastrointestinal adverse events occurred within 7 days after discharging home. All data were analyzed by two independent statistical experts.

### Definitions

1. Feeding intolerance (FI) was defined with the presence of any two of these criteria [15, 20]: (1) decrease, delay, or cease enteral feeds (requirement > four hours) leading to disruption of the patient’s feeding plan, (2) gastric residuals > 50% on a single occasion among infants who received nasogastric feeds, (3) abdominal distension (increase in abdominal girth by > 2 cm from baseline), (4) emesis (requirement over 1 time with brown, bilious, or blood stained gastric aspirates without specific amount), and (5) gross or occult blood in the stool. All criteria should be confirmed by two independent observers.
2. Necrotizing enterocolitis (NEC, Bell stage 2 or 3) was diagnosed based on the presence of one or more of the parenthesized clinical signs (bilious gastric aspirate or emesis, abdominal distention, and occult and/or gross blood in stool (no fissures)) and the presence of at least one of the following three radiographic or sonographic findings: (1) pneumatosis intestinalis, (2) portal vein gas and/or (3) pneumoperitoneum with stage II or higher modified Bell’s staging criteria [21].
3. The criteria for Hypoxic-ischaemic encephalopathy (HIE) diagnosis in our study is formulated by the neonatal group of science, a branch of Chinese Medical Association available on (http://guide.medive.cn/) and meet the following characteristics: (1) signs of fetal distress, as indicated by the cardiotocographic pattern of late decelerations, bradycardia, meconium staining of amniotic fluid and scalp pH < 7.1 or blood lactate levels> 4.8 mmol/L, (2) postnatal distress, as indicated by an Apgar score < 6 at 5 min or pH ≤ 7.00/base deficit≥16 mEq in the umbilical arterial/first postnatal blood from the infant, plus need for neonatal resuscitation for> 3 min, and (3) evidence of neonatal encephalopathy within 6 hours of birth according to the NICHD classification for modified Sarnat staging [18].
4. The criteria for Therapeutic hypothermia (TH) meet one of the follows [19]: (1) Metabolic or mixed acidosis with a pH < 7.0 or base deficit > 12 mmol/L in a sample of umbilical cord blood or any blood obtained within the first hour after birth, (2) meet one of the following: 5-minute Apgar score < 5; ongoing resuscitation (e.g. assisted ventilation, chest compression, or cardiac medications) initiated at birth and continued for at least 10 minutes, and (3) meet the criteria for moderate to severe HIE diagnosis [18].
5. Late-onset bloodstream infection (LOBI, parenteral nutrition analysis) was defined as a blood culture-proven infection with the onset time of symptoms after receiving parenteral nutrition ≥3 days [13].
6. Proven early onset sepsis (EOS) was diagnosed as onset time of the sepsis episode during the first 72 hours of life according to the consensus of Chinese experts (2019 version), which included culture-proven sepsis and clinically proven sepsis [22].
7. Congenital heart defect (CHD) was diagnosed as confirmed structural abnormalities by two-dimensional ultrasonography and was classified as either simple CHD (an isolated and uncomplicated secundum atrial septal defect, patent ductus arteriosus, ventricular septal defect with normal pulmonary vascular resistance, or mild pulmonary stenosis verified) and complex CHD (defects requiring surgery before 12 months of age [23, 24].
8. Newborn hypoglycemia was defined as plasma glucose in the first 48 h of life is < 40 mg/dL (2.2 mmol/l) or after 48 h of life is < 45 mg/dL (2.5 mmol/L) [25].
9. Full enteral feeds was defined as the infant’s total nutrition is from milk as 120 ml/kg/day and termination of total parenteral nutrition with a maximum of 10 ml/kg/day of clear liquids such as NaCl or glucose infusions [4, 26].
10. The initiation time of enteral feeding was defined as the infant first receiving any type of enteral feeding (includes breast milk, artificial formula milk), through any route of administration (includes Oro/Nasogastric tubes, bottles, and breastfeeding) and any milk volume on the first day or after birth [2].
11. Minimal enteral feeding was defined as an enteral intake of breast milk and/or formula, with a small volume of up to 20 mL/kg/day [2].
12. Parenteral nutrition was defined as receiving any type of nutrient solution in any volume through any route of administration (peripheral venous cannula, central venous catheter, or umbilical catheter) for at least 1 day [1].
13. Slow advanced feeding rate was defined as advancing enteral feed volumes at daily increments < 20 ml/kg/day [10].

### Calculation of sample size

The primary comparison will be the difference in the proportion of infants suffering from FI but there is no report about incidence of FI during TH as we know. The sample size justification was based on our hypothesis. Approximately 90–100 babies were admitted to our center with diagnosis of moderate-severe HIE and undergoing TH each year [7, 27]. After considering our exclusion criteria, 20–30% of HIE neonates were expected to be excluded. Hence, as a pilot study and limitation of time in a thesis research we decided to enroll about at least 74 infants with 37 infants in each group. In fact, the sample size in this study was larger than the estimated required number of cases.

### Randomized treatment allocation

Enrollment would occur before the infant’s first enteral feed and after the parents give consent as early as possible after their admission. The eligible infants were randomized to the experimental group (the EEN group) and the control group (the DEN group) using computer-generated numbers, allocated by an independent observer from sealed, opaque, identical envelopes.

### Randomized interventions

Eligible neonates were randomized to receive either early or delayed enteral feeds until the day of reaching full enteral feeds (120 ml/kg. d) or discharge home. The exact same standard feeding procedures (amount, frequency, and advancement of milk) would be followed for infants in the two groups according to the first recommend enteral feeding protocol [28–30] designed for the study in our NICUs for infants, with the one exception that timing of initiation. During the feeding process, if infants were presented FI, the second option of feeding management would be chosen for continued feeds by clinicians (Table 1). All infants were routinely checked abdominal signs mainly including measurement of abdominal circumference and bowel sounds to know their abdomen whether were ready for enteral feeds or not [31]. In the EEN group, when the infants` abdomen examination presented abdominal distention (increase in abdominal girth by > 2 cm from baseline) or abnormal bowel sounds (for 1 minute < 4 times) which often indicated abnormal intestinal function, then enteral feeds would not start at this evaluation time, and the assessment process would be continued every 4–6 hours by two independent clinicians until TH over. Meanwhile, both groups received early parenteral nutrition (Fatty milk, amino acid trace elements, minerals and vitamins) intravenously from day 1–2 onwards. As the volume of milk increased, the volume of PN was decreased gradually. The amount of milk is very small at first, then is gradually increased if the infant tolerated the milk well. When the infants received nasogastric tubes feeding, the nurse would check gastric residuals every feeding. We calculated the amount of milk that could theoretically be achieved within 24 hours as directed by the doctor’s advice (e.g. 5 ml/kg q3h, then the total milk volume is 5*8 = 40 ml/day) per day from the data in corresponding doctor’s advice system records. None of our investigators were involved in the clinical care and treatment of the included infants.

**Table 1:** Recommend enteral feeding protocol at the two groups

Content	EEN group	DEN group
**Initiation time of enteral feeding**	Feed if medically feasible during TH and rewarming	Feed if medically feasible after TH
**Feeding delivery method**	Oral feeding intermittent feeding every 3 hours (20 ~ 30 min) or nasogastric tubes feeding bolus every 3 hours (30 minutes-1 hour) if infants unable to start oral feeds after evaluation of ability for sucking and swallowing daily	Same
**Feeding type of milk at initiation** ^a^	(1) Breast milk or common formula ^b^ if mothers` milk cannot be available (2) Breast milk or deep hydrolyzed protein milk if mothers` milk cannot be available when infants developed FI (second choice)	Same
**Volume of initiation enteral feeding**	10 ~ 20 ml/kg/d	Same
**Speed of feeding advancement** ^a^	(1) 10 ~ 20 ml/kg/d after starting feeds for 3 days and the maximum speed is 30 ml/kg/d (first choice) (2) 5 ~ 10 ml/kg/d for 3 days and the maximum speed is 20 ml/kg/d (second choice) when infants developed FI	Same
**Frequency of feeding advancement** ^a^	(1) Advancement of milk volume each 1 day up to the goal of full enteral feeds (first choice) (2) A short fast if necessary, then advancement of milk volume each 1 ~ 2 days up to the goal of full enteral feeds (second choice) when infants developed FI	Same
**Goal of full enteral feeds**	120 ml/kg/d	Same
**Maximum feeds** ^a^	(1) 150 ~ 160 ml/kg/d (first choice) (2) 140 ~ 150 ml/kg/d (second choice) when infants developed FI	Same

*EEN* Early enteral nutrition, *DEN* Delayed enteral nutrition, *TH* Therapeutic hypothermia, *FI* Feeding intolerance

^a^ All second choice should be only given to infants when clinicians consider them may occur feeding intolerance who cannot continue first choice

^b^ Common formula is defined as formula for term and near term infants (GA ≥ 35 weeks) containing energy as 67 ~ 68 kcal per each 100 ml

The criteria for stopping the trial intervention: (1) The infants appeared to suffer from any serious adverse events for enteral feeds mainly including NEC or obstruction, (2) or the infants developed severe/unstable hypotension [30], or severe pulmonary hemorrhage which lead to stop TH and fasting must last for ≥24 hours.

The criteria for withdrawal from the study: (1) If feeds couldn’t be started during TH/rewarming, then infants belonged to the EEN group would be excluded from the trial. (2) The infants whose hospital stays were shorter than 3 days for abandoned treatment for the opinion of the parent’s decisions with incomplete data would be excluded from the trial too. At all stages it would be made clear to the parents that they would be free to withdraw their infants from the trial at any time, without the need to provide explanation. The infants would be fed following the current standard procedures for HIE directed by the clinical team. (3) The opinion of the treating clinicians for infants had no realistic chance of survival after randomized and was not suitable to start enteral feed.

### Blinding for the study

This was a non-blinded trial; blinding of the treating clinicians, nursing staff, the parents were not possible in clinic practice. Even we these researchers, data evaluators and statisticians would be unblinded once when we saw the time of initiation of feeds. Thus, the medical and nursing teams caring for the infants would be ware of the details of feeding after the infants were randomized to either the EEN group or DEN group.

### Outcome measures

1. Primary outcome to be analyzed was the proportion of infants suffering from FI from trial entry to discharge home.
2. Secondary outcomes to be assessed when infants are discharged home for the first time were: the incidences of late-onset bloodstream infection (parenteral nutrition analysis) and hypoglycemia, survival at neonatal discharge, duration of parenteral nutrition, duration until attainment of full enteral feeds, length of hospital stay and body weight gain.

### Statistical analyses

All data were analyzed using SPSS 26.0 software (IBM, Armonk, New York). Normally distributed continuous data were described as the mean ± standard deviation (M ± SD) and tested by the Student t-test. Skewed data were described as the median and interquartile range and analyzed by the Mann-Whitney U test. Categorical data were shown in percentages and analyzed by the chi-square test or Fisher’s exact test. Kaplan–Meier survival analysis was used to represent time to suffering from FI. A *p*-value< 0.05 was considered statistically significant.

## Results

### Clinical data

**Fig. 1:**
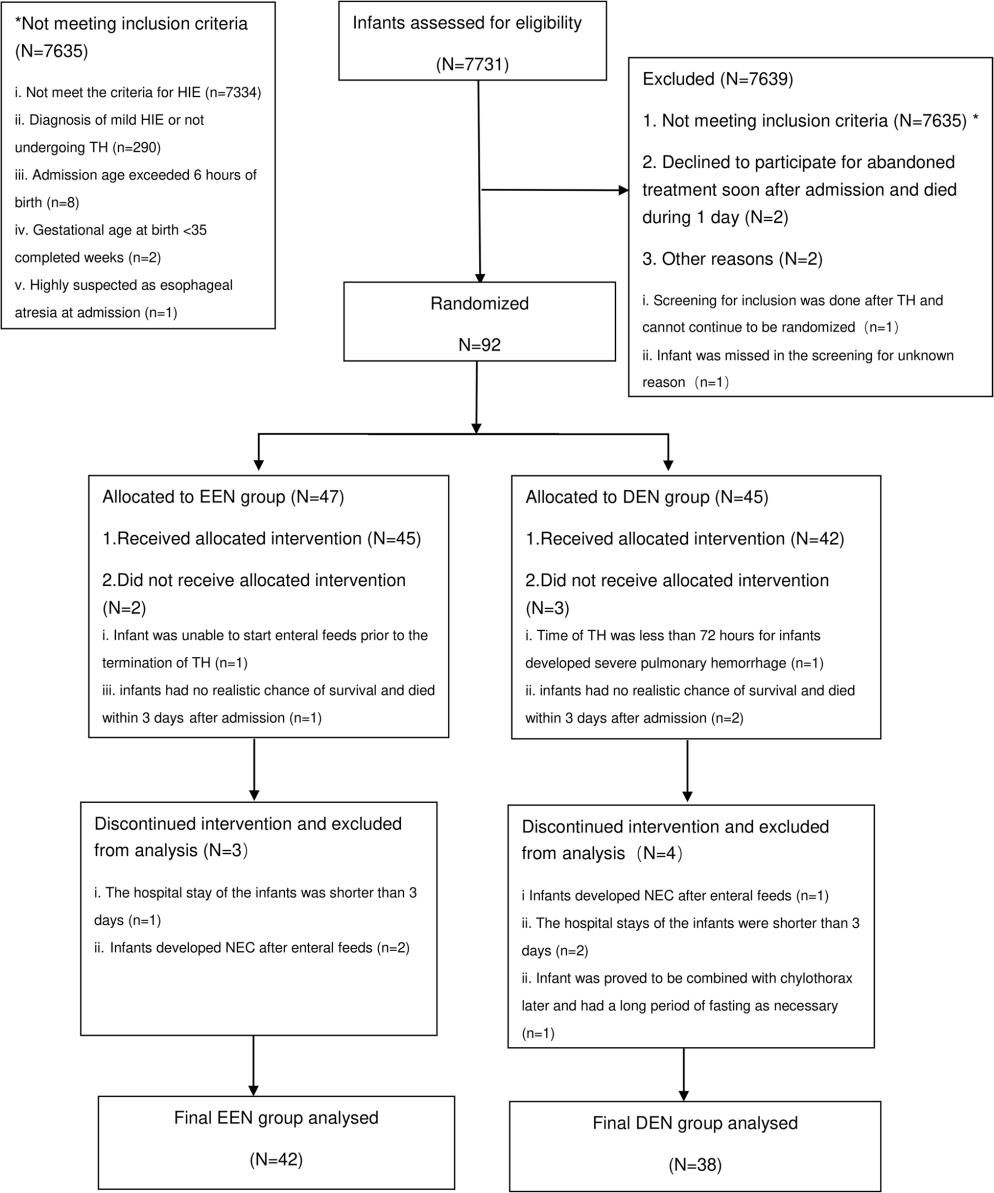
Patient selection flow chart

During the study period, from September 2020 to August 2021, a total of 7731 infants were admitted to our NICUs, and there were 397 (5.14%) infants diagnosed with HIE. Among them 92 (23.17%) cases diagnosed with moderate-severe HIE and undergoing TH were included for the study. Among the eligible neonates, there were 47 and 45 cases who were randomized to the early enteral nutrition group and the delayed enteral nutrition group respectively. There were 12 infants who represented 13.04% of randomized group (total 5 and 7 infants in the EEN and DEN group respectively) were excluded from eventually analyzed with a balance between two groups (x^2^ = 0.785, *p* = 0.376) as follows: (1) 5 infants were excluded prior to initiate the intervention who met the criteria for withdrawal from the study. (2) 7 cases were excluded for discontinuing the intervention who met the criteria for stopping the trial intervention. The 12 infants who were excluded from final analysis had received standard care and therapy for HIE. Among them, first, there were total 3 (3.26%) deaths among two groups with no difference (2.13% vs 4.44% *p* = 0.613): 1 case in the EEN group for multiple organ failure and 2 cases in the DEN group for multiple organ failure and herniation of the brain during 1–3 days after admission, and none of them had started enteral feeds before death. Second, there were also 3 (3.26%) cases developed NEC (Bell stage 2) after enteral feeds among two groups with no difference (4.25% vs 2.22% *p* = 1.000): 2 case in the EEN group and 1 cases in the DEN group. Besides 3 deaths, all cases recovered well and were discharged home according to the clinician’s decisions (Fig. 1).

After randomization, 80 cases who completed the interventions were eventually analyzed. Our hospital is a pediatric tertiary hospital without a delivery room, so infants who were admitted to our NICU all came from other hospitals by emergency cars. All infant’s initial resuscitation for asphyxia were done outside of our NICU, thus many infants cannot obtain complete gas data. Only 46 (57.5%) infants (26 in the EEN group and 20 in the DEN group) could obtain their blood gas data within 1 hour after birth (including umbilical cord gas values) in this study. The severity of HIE or asphyxia (Apgar scores, blood gas and lactate) or complications (CHA and EOS) or therapy (antibiotics and vasoactive drug) which may affect the feeding as confounding factors between the two groups had no differences. A presentation of other demography, complications, laboratory results and adjuvant therapy of neonates were analyzed and found no group differences (all *p* > 0.05) among the variables listed in Tables 2, 3 and 4.

**Table 2:** Comparison of the baseline characteristics at the two groups

	EEN group(***N*** = 42)	DEN group (***N*** = 38)	T/Z/χ2	***p-*** value
Male gender, n (%)	22 (52.38)	22(57.89)	0.245	0.621
Gestational age (weeks) ^a^	39.91 ± 1.14	39.53 ± 1.44	1.320	0.191
Birth weight (kg) ^a^	3.15 ± 0.37	3.15 ± 0.40	−0.007	0.994
Admission age (hours) ^b^	2.00 (2.00,2.00)	2.00 (1.38,2.00)	−0.408	0.683
Gestational age compared with birth weight, n (%)				
Large for GA	1 (2.38)	1 (2.63)	1.404	0.496
Suitable for GA	34 (80.95)	34 (89.47)		
Small for GA	7 (16.67)	3 (2.63)		
EOS, n (%)	9 (21.43)	8 (21.05)	0.002	0.967
Preterm infant (%)	1 (2.38)	1 (2.63)		1.000 ^c^
CHD, n (%)				
No CHD	12(28.57)	17(44.74)	2.256	0.133
Simple CHD	30(71.43)	21(55.26)		
Complex CHD	0(0.00)	0(0.00)		
Sarnat clinical stages of HIE, n (%)				
Moderate	28 (66.67)	25 (65.79)	0.007	0.934
Severe	14 (33.33)	13 (34.21)		
Apgar scores ^a^				
1-min Apgar	5.45 ± 1.95	4.66 ± 2.17	1.723	0.089
5-min Apgar	7.88 ± 1.82	7.03 ± 2.53	1.745	0.085
10-min Apgar	8.83 ± 1.45	8.21 ± 2.11	1.553	0.124
Vaginal delivery	21(50.00)	16(42.11)	0.500	0.479
Meconium stained amniotic fluid	16(38.10)	16(42.11)	0.134	0.715
Premature rupture of membranes	7(16.67)	6(15.79)	0.011	0.915
Intrauterine distress	15 (35.71)	15(39.47)	0.120	0.729
Gestational diabetes	2 (4.76)	3(7.89)		0.664 ^c^

*EOS* Early onset sepsis, *GA* Gestational age, *CHD* Congenital heart defect, *HIE* Hypoxic-ischemic encephalopathy

^a^ Mean and standard deviation

^b^ Median and interquartile range

^c^ Fisher’ exact test

**Table 3:** Comparison of the baseline characteristics at the two groups

	EEN group(***N*** = 42)	DEN group (***N*** = 38)	T/Z/χ2	***p-***value
**Laboratory results**				
Blood gas within 1 hour after birth ^a,b^				
Blood lactic acid (mmol/L)	5.20 (3.40,6.25)	5.25 (3.25,7.63)	0.432	0.666
Base deficit (mmol/L)	−11.15 (− 12.85,-8.18)	−7.40 (− 10.03,-4.55)	−0.986	0.324
pH	7.03 (6.95,7.17)	7.09 (7.00,7.24)	0.888	0.375
Blood gas at admission ^a^				
Blood lactic acid (mmol/L)	5.20 (3.40,6.25)	5.25 (3.25,7.63)	0.106	0.916
Base deficit (mmol/L)	−6.20 (−8.28,-3.68)	−7.40 (−10.03,-4.55)	−1.508	0.132
pH	7.34 (7.31,7.39)	7.32 (7.26,7.42)	−1.201	0.230
Blood sugar at admission (mmol/L) ^b^	4.05 (3.20,5.93)	4.55 (3.70,6.28)	1.364	0.173
Traditional markers of inflammation at admission, n (%)				
Leukocyte< 5 or > 30 (*10^9^/L)	4 (9.52)	2(5.26)		0.678 ^c^
CRP > 8 mg/l	1 (2.38)	0(0.00)		1.000 ^c^
PCT > 0.5 ng/ml	21 (50.00)	19 (50.00)	0.000	1.000
Neutrophil percentage > 80%	7 (16.67)	5 (13.16)	0.193	0.661
**Adjuvant therapy, n (%)**				
PICC for least 3 days	4 (9.52)	5 (13.16)		0.729 ^c^
Antibiotics for least one dose	38 (90.48)	35 (92.11)		1.000 ^c^
Vasoactive drug for least 2 hours	16 (38.10)	10 (26.32)	1.262	0.261

*CRP* C-reactive protein, *PCT* Procalcitonin, *MV* Mechanical ventilation, *PICC* Peripherally inserted central catheters

^a^ Only 46 (57.5%) infants had blood gas data within 1 hour after birth and analyzed for this variable

^b^ Median and interquartile range

^c^ Fisher’ exact test

**Table 4:** Details of enteral feeding at the two groups

		EEN group (***N*** = 42)	DEN group (***N*** = 38)	T/Z/χ2	***p***- value
**Initiation time (days) of enteral feeding** ^a^		2.00 ± 0.91	4.84 ± 0.97	−13.492	< 0.001
**Feeding delivery method, n (%)**					
At initial enteral feeds day	Nasogastric tubes feeding	10(23.81)	12(31.58)	0.604	0.437
	Oral feeding	32(76.19)	26(68.42)		
At discharge home day	Nasogastric tubes feeding	0 (0.00)	0(0.00)		1.000 ^c^
	Oral feeding	42(100.00)	38(100.00)		
**Feeding types of milk, n (%)**					
At initial enteral feeds day	Breastfeeding ^d^	3(7.14)	2(5.26)		1.000 ^c^
	Pure formula feeding ^e^	39 (92.86)	36(94.74)		
At discharge home day	Breastfeeding ^d^	29 (69.05)	21(55.26)	1.617	0.203
	Pure formula feeding ^e^	13 (30.95)	17(44.74)		
**Feeding volume of milk, (ml/kg/d)**					
At initial enteral feeds day ^a^		13.62 ± 2.22	14.43 ± 2.84	−1.412	0.162
At reaching full enteral feeds day ^a,f^		125.79 ± 3.40	124.67 ± 3.98	1.372	0.175
**Speed of feeding advancement**					
From initial to reaching full enteral feeds day ^a,f^		14.79 ± 3.69	15.72 ± 4.38	- 0.979	0.332

^a^ Mean and standard deviation

_b_ Median and interquartile range

^c^ Fisher’ exact test

^d^ Breastfeeding include exclusive and mixed breastfeeding

^e^ Pure formulas feeding include common full-term formula, extensively hydrolyzed protein formula and amino acid formula

^f^ There were only 62 cases (33 cases in the EEN group and 29 in the DEN group) were analyzed with this variable who had reached the goal of full enteral feeding volume before discharged home

### Enteral feeding protocol

Only 62 (77.50%) infants had reached full enteral feeds (33 cases in the EEN group and 29 cases in the DEN group) and another 18 (22.50%) infants (each 9 cases in two groups) received the same feeds management but had not reached the goal of full enteral feeding volume and discharged home earlier for the decision of their family than clinicians recommend. The percentage of infants who had not reached the goal of full enteral feeding volume between the two groups was similar (21.43% vs 23.68%, x^2^ = 0.058, *p* = 0.809). Average length of initiation time of enteral feeding was much shorter in the EEN group compared to the DEN group (2.00 ± 0.91 vs 4.84 ± 0.97 days, x^2^ = − 13.492, *p* < 0.001). The feeding delivery method and feeding types of milk in the groups were similar at initiation and discharge time(*p* > 0.05). The average volume of feeding milk at initial enteral day was found no statistical differences between two groups (13.62 ± 2.22 vs 14.43 ± 2.84 ml/kg. d, T = -1.412, *p* = 0.162). The average milk volume and speed of advancement milk during feeding process between the two groups among infants who had achieved full enteral feeds presented no differences (125.79 ± 3.40 vs 124.67 ± 3.98 ml/kg. d, T = 1.372, *p* = 0.175; 14.79 ± 3.69 vs15.72 ± 4.38 ml/kg. d, T = -0.979, *p* = 0.332 respectively). Besides initial time of enteral feeds, two groups had processed the same feeding method as shown in Table 4.

### Clinical outcome

**Fig. 2:**
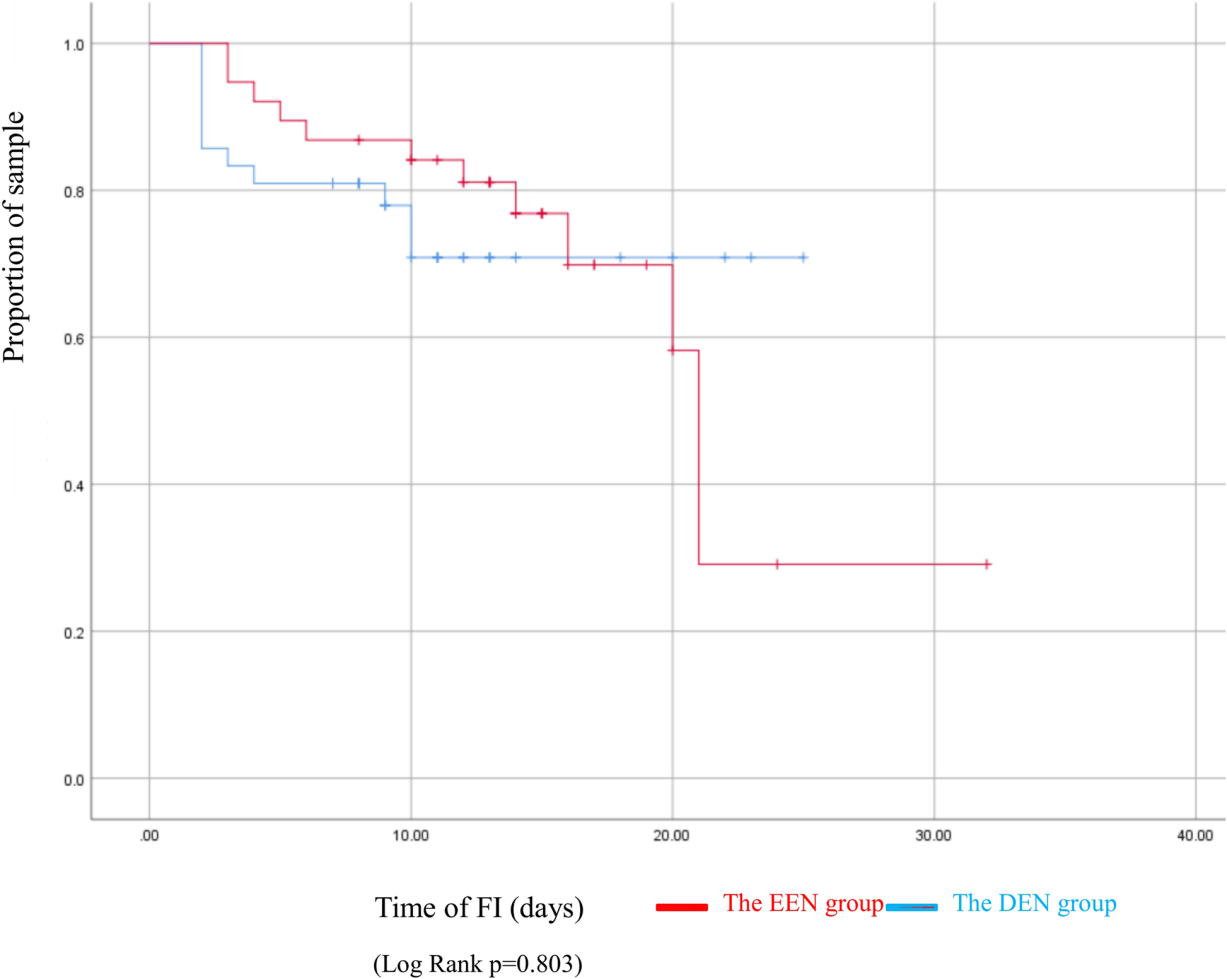
Kaplan Meier curve comparing the feeding intolerance (FI) of the two groups

Primary outcomes: There were total 23 (28.75%) cases developed FI. The incidence of FI was not different in neonates of the EEN group compared to the DEN group (26.19% vs 31.58%, x^2^ = 0.283, *p* = 0.595) (Table 5). No significant difference of incidence of FI was also observed in Kaplan-Meier analysis (Log Rank, x^2^ = 0.062, *p* = 0.803) (Fig. 2).

**Table 5:** Clinical outcomes at the two groups

	EEN group(***N*** = 42)	DEN group (***N*** = 38)	T/Z/χ2	***p***- value
**Primary outcomes**				
Incidence of FI	11 (26.19)	12 (31.58)	0.283	0.595
**Secondary outcomes**				
Incidence of LOBS, n (%)	0(0.0)	2(4.1)		0.222 ^c^
Incidence of hypoglycemia, n (%)	6(14.29)	6(15.79)	0.035	0.851
Duration (days) of PN ^d^	8.81 ± 1.67	10.61 ± 2.06	−4.299	< 0.001
Duration (days) from initiation to reaching full enteral feeds ^a,d^	9.91 ± 1.88	12.24 ± 2.50	−4.182	< 0.001
Length (days) of hospital stay ^a^	12.55 ± 4.57	16.47 ± 5.27	−3.566	0.001
Weight (kg) at discharge home day ^a^	3.24 ± 0.40	3.28 ± 0.41	−0.393	0.695
Weight gain (g/d) from birth to discharge home day	8.41 (−1.69,15.79)	8.22 (0.92,14.78)	0.101	0.919

*NEC* Necrotizing enterocolitis, *FI* Feeding intolerance, *PN* Parenteral nutrition, *LOBI* Late-onset bloodstream infection

^a^ Mean and standard deviation

^b^ Median and interquartile range

^c^ Fisher’ exact test

^d^ There were only 62 cases (33 cases in the EEN group and 29 in the DEN group) were analyzed with this variable who had reached the goal of full enteral feeding volume before discharged home

Secondary outcomes: There was no case died and suffering from LOBS among final analyzed infants. There was also found no difference for the incidence of hypoglycemia (14.29% vs 15.79%, x2 = 0.035, *p* = 0.851). The average times of parenteral nutrition, reaching full enteral feeds and hospital stay were shorter in the EEN group compared with the DEN group with differences (8.81 ± 1.67 vs 10.61 ± 2.06 days, T = -4.299,* p* < 0.001; 9.91 ± 1.88 vs 12.24 ± 2.50 days, T = -4.182, *p* < 0.001; 12.55 ± 4.57 vs 16.47 ± 5.27 days = − 3.566, *p* = 0.001 respectively). Weight at discharge day and weight gain (between birth day and discharge home day) were found no differences [3.24 ± 0.40 vs 3.28 ± 0.41 kg, T = -0.393, *p* = 0.695; 8.41 (− 1.69,15.79) vs 8.22 (0.92,14.78) g/d, Z = 0.101, *p* = 0.919 respectively].

Follow up among the cases who had not reached full enteral feeds: 7 days after these patients discharged home, a telephone call was done by one researcher to know the outcome at follow up day. There were 17 cases (94.44%) successfully followed up and 1 case (5.56%) could not be connected in the EEN group. Further comparison was not done between the two groups for so small sample. There was only 1 case in the EEN group suffered from FI who presented visible blood stool and was readmitted to the hospital at 14 days old. After his readmission, we have excluded gastrointestinal infection as norovirus or rotavirus according to his laboratory results were normal and fasting for 2 days’ treatment is effective, and he was discharged home after 5 days of hospital stay. No one of the infants developed NEC or death after discharging to home. Most of them (94.12%) took breastfeeding at home and all achieved full enteral feeds.

## Discussion

Adjunctive interventions for infants with HIE undergoing therapeutic hypothermia is an important area of research to optimize outcomes for this high-risk group of infants. There is limited data to guide nutritional support. Therefore, our RCT is encouraging to undertaken even with a small sample size. Infants were randomized to initiation of enteral feeds either during or after therapeutic hypothermia/rewarming and a feeding guideline was provided.

The time to start feeding was according to the randomized group but feeds were only initiated when participating infants were assessed with normal intestinal function by abdominal examination. Initiation time of enteral feeding in the present study was 2.00 ± 0.91 days in the EEN group while in the DEN group was 4.84 ± 0.97 days similar as Chang et al. [3]. This showed that most clinicians had chosen to start feeding at the second day according to their judgments of the infants` abdomen. Besides initial time of enteral feeds, two groups had processed the similar feeding method. The milk volume of enteral feeds given at the initiation day in both two groups were minimal volume < 20 ml/kg/day (13.62 ± 2.22 vs 14.43 ± 2.84 ml/kg. d, *p* = 0.162) and the speed of advancement milk volume in both two groups were slow rates < 20 ml/kg/day (14.79 ± 3.69 vs15.72 ± 4.38 ml/kg. d, *p* = 0.332) (10,30). Previous studies suggest that minimal enteral feeding for term and near-term infants with moderate or severe HIE who receive whole body hypothermia is feasible and not associated with significant complications [10]. On the other hand, faster increases in milk feed volumes may increase the risk of NEC, which may provoke feeding intolerance or gut dysfunction. No advantage of increasing feeds at faster or slower rates was identified by some observational data [29]. Therefore, the feeding strategy suggested in our study recommended minimal enteral feeding and slow speed of increasing milk feeds to ensure the safety of neonates receiving TH.

The incidence of NEC in neonatal HIE is much lower [13] which even reported as no one developed NEC according to previous reports [1–6]. Because the infants in these study almost full term or near term infants, as in our study there were only two preterm infants, therefor, NEC became a rare disease with very low morbidity (0–0.5%), so it is hard to recruit sufficient patients to participate our research to show differences in groups for NEC. In our trial, there were 3 (3.26%) NEC among two groups with no difference (4.25% vs 2.22% *p* = 1.000) who were excluded from the final analysis.

Although there was no death in the final analyzed infants, but there were 3 (3.26%) deaths after randomization without difference between the two groups (2.13% vs 4.44% *p* = 0.613). No one died for feeds because no one started feeding. The incidence of hypoglycemia and weight gain in both two groups was similar too. These findings were consistent with some retrospective study [1–7, 13]. There was also no case suffering from LOBS in our study and some previous reports in neonates with HIE have not found an increased incidence of bacteremia during or following hypothermia therapy and which was consistent with our result [19].

Our study confirmed that FI was very frequent in the infants with HIE. It had been proved that 28.75% of infants including in our study developed feeding intolerance with a similar morbidity among common preterm infants 16–29% [32, 33]. And early enteral nutrition did not increase the risk of FI compared with delayed enteral feeding (26.19% vs 31.58%, x^2^ = 0.283, *p* = 0.595; Log Rank, x^2^ = 0.062, *p* = 0.803), which had never been reported before. Not only NEC but also FI should be observed among infants with HIE when undergoing TH for a high morbidity of FI in these population. This complication is relevant for its potential correlation with the development of necrotizing enterocolitis (NEC) [34]. One of the risk factors for the development of feeding intolerance is the impairment of mesenteric perfusion and oxygenation [20, 34], which may both occurred in the infants with HIE and perinatal asphyxia. Therefore, FI is common in this population and has a high incidence.

The short-outcomes between the two groups among the 18 cases who had not achieved the full feeds were similar and only 1 case in the EEN group developed FI after discharging to home. Our results indicated that there were no differences in feeding intolerance or other gastrointestinal adverse events or serious complications between groups. Therefore, we considered that the early enteral nutrition possibly proved to be feasible and safe as delayed enteral nutrition with minimal enteral feeding and slow advance feeding rate.

The average time of PN, reaching full enteral feeds and hospital stay were shorter in the EEN group compared with the DEN group according to our findings, which was similar with Chang et al. [3] and Gale C et al. [13]. The possible reasons may be these: First, infants delayed enteral feeding may cause clinicians increase worry about they not yet give the infants enough time to reach the goal of feeding, so they often stop PN later and allow the infants to discharge home later. Second, the earlier the enteral feeds were started for the infants, the more likely to achieve the target feeding goal with similar feeds method when compared the EEN group to the DEN group. Long-term parenteral nutrition may increase the risk of serious systemic infections, intravenous nutrition-related cholestasis, and damage to the intestinal immunity [35]. On the other hand, long-term hospital stay may lead to more cost as well as increase mother-child separation time. So early enteral nutrition presents some advantages which can shorten these time compared with delayed enteral nutrition.

Feeding strategies during TH are an important area, but so far no RCT studies have been conducted. Why? Maybe there are many difficulties to do it. This trial is also about the feasibility of a feeding intervention as well efficacy. First, there were 12/92 (13.04%) cases of total randomized group could not initiate or continue intervention. The proportion that could not be randomly intervened in the population was large and made the appropriate sample in our study further decreased. Because there is no RCTs before, it is not safe to conduct multiply studies at present in order to include enough cases. Second, in clinic practice, require all clinicians to deal with infants 100% according to a standard protocol would be very difficult to achieve especially when infants developed FI. We allowed two choices of the recommend enteral feeding protocol (Table 1) in the study based on the experience of clinicians in practice to resolve this problem. Third, parental compliance in the study may vary largely. In our study, there were 18 (22.50%) cases were discharged home earlier for the decision of their family than clinicians recommend to achieve full enteral feeds who needed to be follow up to know the outcomes. It was possible to lose complete data if parents could not be connected such as our study had lost 1 case (5.56%) to follow up. Although the sample size in this trail was much small, it had proved the feasibility of a feeding intervention as we descried.

The main strength of our study is that it is the first RCT about enteral nutrition among patients with HIE undergoing TH. Our study had proved the feasibility and safety of early enteral nutrition for HIE compared with delayed enteral nutrition. And in this study, we also examined the incidence of FI in the population for the first time. This trial had also proved the feasibility of a feeding intervention in neonatal HIE.

This study has some limitations. First, the study not achieved double-blindness. Although clinicians in two groups have adapted their feeding process from our recommended feeding strategies, differences in attitudes and practices might explain differences in time to stop PN, as well as length of hospital stay. These limits the generalizability of our findings. There were only 3 deaths in this cohort and excluded from final analysis which thus limited in terms of evaluating death risk for such a small sample size. Therefore, this study can be further expanded to include more infants and will be designed to assess mortality in the population.

## Conclusion

Compared with delayed enteral nutrition, introduction of early enteral nutrition according to a recommend feeding strategy (including minimal enteral feeding and slow advance feeding rate) for patients with neonatal hypoxic-ischemic encephalopathy (HIE) undergoing TH may be feasible and safe.FI is frequent in this high-risk group of infants which should not be ignored during feeding process.

## Data Availability

The dataset used and/or analyzed during the current study are available from the corresponding author on reasonable request. All data generated or analyzed during this study are included in this published article. Proposals should be submitted to 21277201@qq.com.

## Abbreviations

TH: Therapeutic hypothermia
HIE: Hypoxic-ischemic encephalopathy
NEC: Necrotizing enterocolitis
FI: Feeding intolerance
NICUs: Neonatal intensive care units
EEN: Early enteral nutrition
DEN: Delayed enteral nutrition
PN: Parenteral nutrition
RCT: Randomised controlled trial
CHD: Congenital heart defect
UK: The United Kingdom of Great Britain and Northern Ireland
CRP: C-reactive protein
PCT: Procalcitonin
MV: Mechanical ventilation
PICC: Peripherally inserted central catheters
EOS: Early onset sepsis
GA: Gestational age
LOBI: Late-onset bloodstream infection
